# A Mechanogenetic Model of Exercise-Induced Pulmonary Haemorrhage in the Thoroughbred Horse

**DOI:** 10.3390/genes10110880

**Published:** 2019-11-01

**Authors:** Sarah Blott, Hannah Cunningham, Laurène Malkowski, Alexandra Brown, Cyril Rauch

**Affiliations:** 1 School of Veterinary Medicine and Science, University of Nottingham, Sutton Bonington LE12 5RD, UK; 2 Animal Health Trust, Lanwades Park, Kentford, Newmarket, Suffolk CB8 7UU, UK

**Keywords:** exercise-induced pulmonary haemorrhage, horse, genome-wide association, capillary fracture, cell–cell adhesion, cell cytoskeleton stiffness, blood flow

## Abstract

Exercise-induced pulmonary haemorrhage (EIPH) occurs in horses performing high-intensity athletic activity. The application of physics principles to derive a ‘physical model’, which is coherent with existing physiology and cell biology data, shows that critical parameters for capillary rupture are cell–cell adhesion and cell stiffness (cytoskeleton organisation). Specifically, length of fracture in the capillary is a ratio between the energy involved in cell–cell adhesion and the stiffness of cells suggesting that if the adhesion diminishes and/or that the stiffness of cells increases EIPH is more likely to occur. To identify genes associated with relevant cellular or physiological phenotypes, the physical model was used in a post-genome-wide association study (GWAS) to define gene sets associated with the model parameters. The primary study was a GWAS of EIPH where the phenotype was based on weekly tracheal wash samples collected over a two-year period from 72 horses in a flat race training yard. The EIPH phenotype was determined from cytological analysis of the tracheal wash samples, by scoring for the presence of red blood cells and haemosiderophages. Genotyping was performed using the Illumina Equine SNP50 BeadChip and analysed using linear regression in PLINK. Genes within significant genome regions were selected for sets based on their GeneOntology biological process, and analysed using fastBAT. The gene set analysis showed that genes associated with cell stiffness (cytoskeleton organisation) and blood flow have the most significant impact on EIPH risk.

## 1. Introduction

Exercise-induced pulmonary haemorrhage (EIPH) is frequently identified in horses performing high-intensity athletic activity. During exercise the pressure in the pulmonary capillaries reaches at least 80 mmHg [1], which is sufficient to cause pulmonary capillary stress failure [2,3]. Additionally, high transmural pressure (difference between the positive intraluminal and negative alveolar pressures) causes rupture of the blood-gas barrier [4]. The cause of bleeding is capillary failure; bleeding stops when the pressure is reduced [5] but lung lesions remain in horses with EIPH suggesting the condition is due to more than simple stress failure [6,7,8]. During exercise, raised left atrial pressure causes blood to back up into the lungs leading to an increase in pulmonary capillary pressure [9]. As a result, in EIPH lung lesions the small pulmonary veins (100–200 µm outer diameter) are remodelled through the build-up of adventitial collagen and hyperplasia of smooth muscle [5] which can lead to a significant reduction in the vascular lumen [6,7,8].

EIPH lung lesions have been observed in unraced Thoroughbreds in training [10], suggesting that venous remodelling may begin when horses first enter training. It has been hypothesised that the frequency and duration of ‘high-pressure events (HPE)’—gallops during training or races—and the horse’s genetic susceptibility to vascular weakness will determine the degree of venous remodelling seen in an individual horse [5]. This hypothesis is supported by observations that pulmonary veins are stiffer in raced than unraced horses [11], and that number of lifetime starts rather than age correlates with EIPH [12]. Long term prognosis for horses with EIPH is poor, there is no cure and the only way to allow horses to recover is by giving them enough rest time between races or by retiring them [13].

Historically there have been many documented references to racehorses who were ‘bleeders’. The earliest recorded was a horse called ‘Bartlett’s Childers’ (born 1716), who was the great-grandsire of the stallion ‘Eclipse’, others have included ‘Herod’ (born 1758) and ‘Hermit’ (born 1864). Amongst modern-day Thoroughbred sires, more than 95% can trace their ancestry to ‘Eclipse’ [14]. The heritability of epistaxis (when blood is visible at the nostrils) in UK, Australian, South African and Hong Kong Thoroughbreds has been evaluated based on pedigree analysis, and estimated at 0.3–0.5 [15,16,17] providing clear evidence of a genetic basis to EIPH risk, although the aetiology is complex.

Genome-wide association studies (GWAS) have played a key role in the search for genetic variants associated with complex traits and several secondary analysis methods have been proposed for fine mapping of causal variants within an associated genome region, or for prioritizing variants based on functional annotation. Complex traits involve multiple genes and the impossibility of considering single variants without the context of their genetic background has led to the development of several methods that explore the combined effects of many genes. Gene set analyses are a popular approach, as they can give insight into disease mechanisms and functional context for individual single nucleotide polymorphism (SNP) associations [18]. In this study we show how a physics-inspired model of EIPH can be used to define gene sets based on parameters having a significant influence on the model, namely cell–cell adhesion, cell stiffness (cytoskeleton organisation) and blood flow. This enables gene sets to be refined according to biological functions which specifically affect the physical phenotype.

## 2. Materials and Methods

### 2.1. Tracheal Wash Sampling

Tracheal wash samples were collected from 106 thoroughbred horses in a flat racing training yard by the same veterinary surgeon. Samples were collected on a weekly basis from March 2009 to November 2010, with 41 horses sampled in 2009, 42 horses sampled in 2010 and 23 horses sampled in both 2009 and 2010. During the sampling period, all horses were in training and raced at regular intervals. The tracheal wash (TW) samples were obtained transendoscopically post-exercise during training, using the biopsy probe of a flexible endoscope. Sample collection and handling protocols were reviewed and approved by the Ethics Committee at the Animal Health Trust (AHT01-2008). Only horses that were sampled on four or more occasions during the sampling period and whose genotyping data passed genotyping quality control were included in this study. This left a total of 72 horses in the study, 32 males and 40 females.

### 2.2. Tracheal Wash Cytology

Tracheal wash samples were formalin-fixed at the point of collection. The wash sample was centrifuged, and a portion of the cell pellet was pipetted onto a Poly-L-Lysine glass slide and smeared. In samples where a reasonably sized pellet was not visible after centrifugation, slides were prepared using cytospin centrifugation. All slides were air-dried, stained using Haematoxylin and Eosin stains and examined using light microscopy. Each sample was evaluated for the presence of epithelial cells, macrophages, lymphocytes, eosinophils, haemosiderophages (HF), erythrocytes or red blood cells (RBC), squames, mucus, fungi, bacteria, plant material and debris. Each of these cell types or characteristics was assigned a score out of three indicating a relative density, with a score of 0 equating to none, score of 1 to occasional, score of 2 to moderate and a score of 3 to high (see Appendix A for cytology images). Overall cell density was scored out of 3; a score of 0 equated to low/medium-low, a score of 1 to medium, a score of 2 to medium-high and a score of 3 to high.

### 2.3. EIPH Phenotype

EIPH is defined as blood in the airways after exercise and may be detected by tracheobronchoscopic examination and/or by enumerating red blood cells or haemosiderophages in cytology samples from tracheal wash (TW) or bronchoalveolar lavage (BAL) fluid [19]. BAL requires sedation and local anaesthesia of the horse, as the endoscope is passed further than the trachea and into a subsegmental bronchus [20]. The need for sedation and local anaesthesia means that the routine use of BAL on horses engaged in both training exercise and racing is not acceptable to trainers, hence this study was based on routine (weekly) tracheoendoscopic examination and TW sampling rather than BAL. The occurrence of EIPH was determined by cytological analysis of the TW samples to score the presence of red blood cells and haemosiderophages (macrophages which have phagocytized red blood cells in the alveolar space or airways). Red blood cells are detectable immediately post-exercise in BAL fluid, while the percentage of haemosiderophages significantly increases one week after exercise returning to pre-exercise levels within three weeks [21]. Although TW does not directly correlate with BAL cytology [20], the numbers of haemosiderophages may be used to detect EIPH in TW samples [22]. While the precise timing of haemorrhage is difficult to determine from TW samples, an increased frequency of haemosiderophage score during the time period of sampling indicates persistent episodes of bleeding after training and racing. In this study, the EIPH phenotype was obtained as a frequency score for each horse calculated as the sum of the HF scores (>1) over the series of tracheal wash samples taken on that individual divided by the total number of samples in the series.

### 2.4. Horse Race Record

The number of races starts in both 2009 and 2010, dates of first and last race start for both 2009 and 2010, gender and date of birth for each horse was obtained by searching the Racing Post Online database (http://www.racingpost.co.uk). This information was used to calculate the age of each horse at first race start for both 2009 and 2010, and the age of each horse when the first tracheal wash sample was collected. The age of the horse at first race start and age at first tracheal wash sampling was significantly correlated (r = 0.895). The number of days between the last race start and each tracheal wash sampling was also calculated.

### 2.5. DNA Extraction and Quantification

Blood samples from each horse were collected for routine haematology testing as part of the animal’s training regime by a veterinary surgeon, and residual samples were used for DNA extraction. DNA was extracted using nucleon BACC DNA extraction kits. Samples were quantified in duplicate using Quant_iT PicoGreen dsDNA kits (Invitrogen Carlsbad, CA, USA) and 10% of the samples were run on a 1% agarose gel to check for the presence of high molecular weight DNA. DNA aliquots were adjusted to a concentration of 70 ng/uL for genotyping.

### 2.6. Single Nucleotide Polymorphism (SNP) Genotyping and Quality Control

Samples were genotyped using the Equine SNP50 BeadChip (Illumina, San Diego, CA, USA). This array consists of 54,602 SNP (single nucleotide polymorphisms) assays, all of which are derived from the EquCab2.0 SNP collection (http://www.broadinstitute.org/mammals/horse). SNPs are evenly distributed throughout the equine genome, with an average density of one SNP per 43.2 kb.

The genotyping data were analysed with GenomeStudio software (Illumina, San Diego, CA, USA) using a cluster file generated from a dataset of 1342 Thoroughbred samples previously genotyped with the Equine SNP50 BeadChip. SNPs with low intensity data (190 SNPs), with inadequately defined clusters (1265 SNPs), with call rates less than 98% (2279 SNPs), where the heterozygote cluster was insufficiently separated from the homozygote clusters (297 SNPs), where genotypes differed significantly from Hardy–Weinberg equilibrium (119 SNPs) and where X chromosomes SNPs were heterozygous in males (52 SNPs) were removed. Additionally, SNPs for which more than 10% of genotypes were missing, markers that failed the Hardy–Weinberg equilibrium test (*p* < 0.001), and markers with a minor allele frequency (MAF) less than 2% were removed leaving a total of 43,465 SNPs in the analysis.

### 2.7. Population Structure

**Figure 1:**
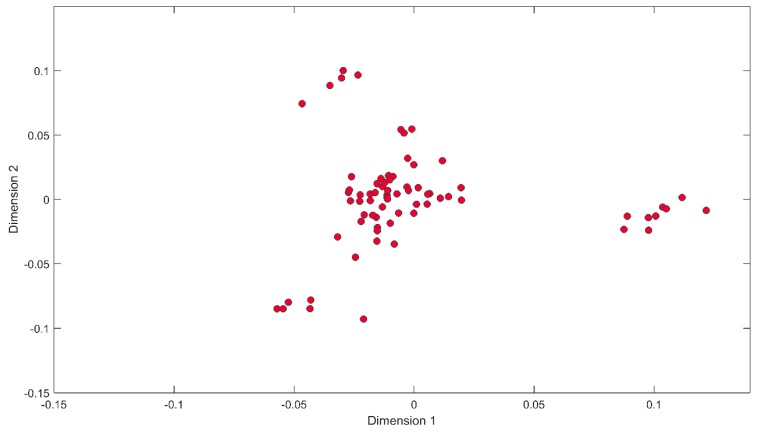
Multidimensional scaling (MDS) plot of genetic relationship among all individuals, calculated from the whole genome single nucleotide polymorphism (SNP) genotypes. The outlying clusters represent three half-sibling families.

Possible population stratification was assessed by calculating identity-by-state (IBS) sharing among all pairs of individuals. A permutation test for between-group IBS differences was carried out in PLINK v1.07 [23]. Groups were defined as horses with HF frequency score greater than zero (EIPH affected) or equal to zero (unaffected). The IBS relationships among individuals were visualised using multidimensional scaling to plot the genetic relationship matrix (Figure 1).

### 2.8. Linear Regression Association Analysis

An association analysis to identify SNPs significantly associated with EIPH was carried out using the linear regression option in PLINK v1.07 [23]. Each SNP is analysed independently (single SNP analysis), with a regression coefficient significantly different from zero indicating a relationship between the genotype at that SNP and the phenotype. A *t*-test is used to test whether or not the coefficient is significantly different from zero. Linear regression analysis also allows for the testing of disease trait SNP associations along with interaction with defined co-variates. Covariates incorporated into the association analysis included gender, age of horse at first race start within the period of time when tracheal wash sampling occurred, period(s) of time when tracheal wash sampling occurred and the average number of days to last race start before tracheal wash sampling occurred. A Bonferroni correction was applied to adjust the significance of *p*-values to account for multiple testing; in this case, 43,417 SNPs were tested. A *p*-value was considered to be significant when *p* < 1.15 × 10^−6^.

### 2.9. Estimation of the Genetic Variance Explained by Individual Chromosome SNPs

Restricted maximum likelihood (REML) analysis with the GCTA program [24] was used to obtain estimates of the genetic variance explained by SNPs on individual chromosomes.

### 2.10. Gene Set Analysis

The physical model of EIPH described in Appendix B shows that sustained bleeding will occur when capillary fracture reaches a critical length, the length being determined by the ratio between the energy involved in cell–cell adhesion and the stiffness (cytoskeleton organisation) of cells. Genetic variants that decrease cell–cell adhesion or increase cell stiffness are, therefore, likely to enhance susceptibility to EIPH. The model also suggests that factors modulating blood flow, such as a reduction in blood vessel diameter or an increase in cardiac output, would increase susceptibility to EIPH. The key parameters modulating risk are, therefore, cell–cell adhesion, cell stiffness and blood flow. To identify whether genetic variants identified through the GWAS significantly affected these parameters a gene set analysis was carried out, assigning SNPs from genome regions significantly associated with EIPH to gene sets based on their proximity to known or predicted genes with functions linked to these parameters.

Genes in the genome regions demarcated by the top 20 GWAS SNPs ranked on *p*-value were identified in the Ensembl genome browser (www.ensembl.org), and gene annotations were identified by comparison with the syntenic human regions. Gene function analysis and assignment to Gene Ontology (GO) classifications were carried out using DAVID [25] and Amigo2 [26]. Based on the prior information provided by the physics model, and using GO classifications as gene function, genes were grouped into sets with functions relating to (i) cell–cell adhesion (ii) cytoskeleton organisation (cell stiffness) and (iii) blood flow. For comparison gene sets representing (iv) disease biomarkers (stroke or hypertension) and (v) random background markers (genes within the significant GWAS genome regions but having functions unrelated to the parameters of interest) were also constructed. The GO terms used to classify the gene sets are shown in the Supplementary File: Table S1 Gene Ontology Terms.

For each gene, the chromosome position, gene start and end positions were identified using Ensembl BioMart [27]. SNPs on the Equine SNP50 BeadChip were then assigned to genes within the gene sets if they were located within the gene boundaries (end-start), or within 5 kb or within 20 kb of the gene start or end.

Gene set analysis was carried out using fast set-based association analysis (fastBAT) [28]. SNPs in genes that appeared in the intersect between sets (i) cell–cell adhesion, (ii) cytoskeleton organisation (cell stiffness) and (iii) blood flow were further analysed for haplotype associations and SNP–SNP epistatic interactions using PLINK v1.07 [23].

## 3. Results

### 3.1. Population Structure

All sampled individuals came from bloodlines which predominantly represent the UK Thoroughbred population. Figure 1 shows the majority of individuals were in a central cluster, with three outlying clusters representing three separate sire families. All three of these families trace back to a single sire within three or four generations. The structure seen in the data, therefore, represents close family structure within the breed. There was no significant difference in IBS between groups (horse with HF scores > 1 were defined as cases, all others as controls), with the proportion of variance in IBS between groups = 0.0002, confirming there is no significant stratification within this population.

### 3.2. Genome-Wide Association Analysis (GWAS)

**Figure 2:**
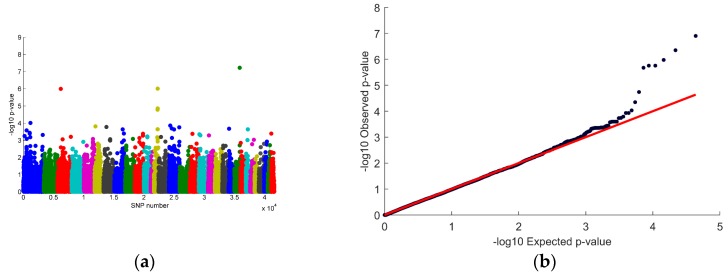
Genome-wide association study results: (**a**) Manhattan plot showing the –log_10_ (*p*-values) for relative sum of haemosiderophage frequency (HF) score > 1 plotted against SNPs colour coded by chromosome; (**b**) Quantile–quantile (QQ) plot of expected versus observed *p*-values with a genomic inflation factor (λ) = 0.993.

Genome-wide significant single nucleotide polymorphisms (SNPs), after adjusting the *p*-values for multiple testing (*p* < 1.15 × 10^−6^), were found on chromosomes 3, 13 and 23 for haemosiderophage frequency (number of times HF score > 1). Suggestive signals for haemosiderophage frequency (HF), not quite reaching genome-wide significance, were found for genome regions on chromosomes 1, 14 and 25. Figure 2 illustrates the genome-wide association results for all SNPs. Appendix C shows the top 20 SNPs from the GWAS, ranked by smallest to largest *p*-value.

SNP genetic variances estimated for each chromosome are listed in the Supplementary file: Table S2 SNP based Estimates of Genetic Variance. Genetic variances significantly different from zero were identified on chromosomes 13, 19 and 20.

### 3.3. Gene Set Analysis

Table 1 shows the GWAS genome regions from which gene lists were identified. The GAD_Disease, Gene Ontology terms, KEGG Pathway and UP Keywords associated with all genes identified in the significant genome regions are listed in the Supplementary file: Table S3 EIPH Top 20 SNPs Gene Functions. Gene sets were constructed based on these terms, in total six sets were tested representing (1) cell–cell adhesion (2) cytoskeleton (cell stiffness) (3) blood flow (4) disease biomarkers for stroke (5) disease biomarkers for hypertension and (6) random genes in the significant genome regions with functions unrelated to categories (1)–(5). The gene names and symbols for all genes contained within gene sets (1)–(5), and the intersections among the gene sets are given in the Supplementary file: Table S4 Gene Set Lists.

Results of the fast set-based association analysis (fastBAT) [28] gene set analysis are summarized in Table 2, Table 3 and Table 4.

When SNPs were restricted to either those positioned within gene boundaries or up to 20 kb from the gene boundaries the significant gene sets were those with functions related to cytoskeleton (cell stiffness), blood flow and biomarkers for hypertension. There was intersection of gene content among the gene sets, with 5 genes (*ABAT*, *CYFIP2*, *EMP2*, *FN1*, *TEK*) being common to cell–cell adhesion, cytoskeleton organisation and blood flow, 9 genes (*AAMP*, *ABAT*, *CYFIP2*, *EMP2*, *FN1*, *KCNMA1*, *RAPIGDS1*, *SGCD*, *TEK*) intersecting between cytoskeleton organisation and blood flow, 14 genes (*ABAT*, *ADGRE2*, *B4GALT1*, *CDH13*, *CYFIP2*, *DLG5*, *EMCN*, *EMP2*, *FN1*, *FOXF1*, *IL12B*, *PLAU*, *PPP3CA*, *TEK*) between cell–cell adhesion and blood flow and 11 genes (*ABAT*, *ARPC2*, *CHMP5*, *CYFIP2*, *EMP2*, *FN1*, *SOCS1*, *TEK*, *TNS1*, *VCL*, *WHRN*) between cell–cell adhesion and cytoskeleton organisation.

The five genes which have functions related to all three physical model parameters (cell–cell adhesion, cytoskeleton organisation or cell stiffness and blood flow) are potentially important candidate genes influencing EIPH risk. *ABAT* (4 aminobutyrate aminotransferase) is a mitochondrial gene involved in the regulation of blood pressure and response to hypoxia. *CYFIP2* (cytoplasmic FMR1-interacting protein 2) forms part of the VEGFA–VEGFR2 pathway and is associated in humans with susceptibility to Kawasaki disease and coronary artery lesions [29], interacting with *PDE2A* so that high-risk allele combinations account for 67% of cases in this human population. *EMP2* (epithelial membrane protein 2) regulates blood vessel endothelial cell migration and angiogenesis by regulating VEGF protein expression through *PTK2* activation [30] and plays a role in the induction of VEGFA via a HIF1A-dependent pathway [31]. *FN1* (Fibronectin) is involved in cell adhesion, cell motility, wound healing and maintenance of cell shape. *TEK* (angiopoietin-1 receptor) regulates angiogenesis and endothelial cell survival, and is associated with pulmonary hypertension [32] and cutanomucosal venous malformation in humans. *ABAT*, *CYFIP2*, *FN1* and *TEK* are all expressed in vascular and lung tissue, *EMP2*, *FN1* and *TEK* are expressed in tracheal tissue (UniGene cDNA sources). Four further genes intersect between cytoskeleton and blood flow: *AAMP* (angio-associated migratory cell protein) is associated with angiogenesis and has potential roles in endothelial tube formation and the migration of endothelial cells, *KCNMA1* (potassium calcium-activated channel subfamily M α 1), *RAP1GDS1* (Rap1 GTPase-GDP dissociation stimulator 1) and *SGCD* (sarcoglycan delta) are associated with total anomalous pulmonary venous return [33].

**Figure 3:**
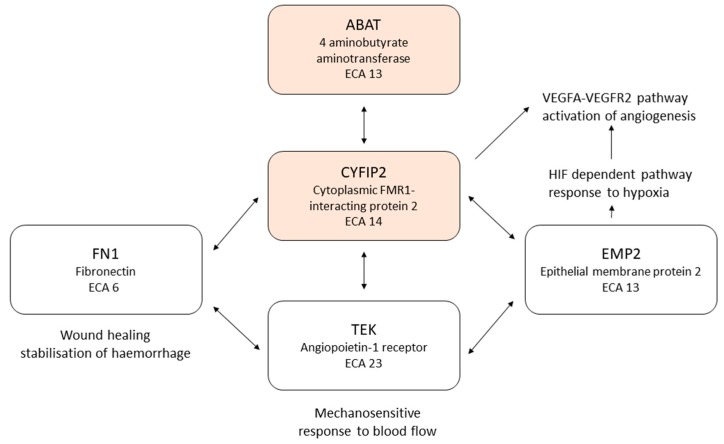
Schematic diagram of the epistatic relationships among SNPs in the genes *ABAT*, *CYFIP2*, *EMP2*, *FN1* and *TEK*. Genes are represented as boxes, with the arrows showing significant epistatic interactions between them. The highlighted (red) boxes are the genes at which haplotypes are significantly associated with the exercise-induced pulmonary haemorrhage (EIPH) phenotype (Table 5).

The analysis of epistatic (gene–gene) interactions among the five genes which have roles in all three biological parameters or functions showed significant (*p* < 0.05) interactions between six SNPs representing all five genes (Appendix D). SNPs in *EMP2*, *ABAT* and *FN1* had significant epistatic interaction with SNPs in *CYFIP2* and *TEK*, and there was also interaction between SNPs in *CYFIP2* and *TEK*. Figure 3 illustrates the epistatic relationships among SNPs in the five genes. Haplotype analysis of SNPs within these five genes showed the haplotypes mostly significantly associated with the EIPH phenotype were in *ABAT* on chromosome 13 and *CYFIP2* on chromosome 14 (Table 5). These results suggest that the interactions between the genotypes at *ABAT*, *EMP2*, *CYFIP2*, *FN1* and *TEK* play an important role in determining EIPH risk.

## 4. Discussion

The initial GWAS analysis identified three genome-wide significant regions associated with haemosiderophage score on chromosomes 3, 13 and 23, with the size of the regions ranging between 1–16 Mb. Altogether, the top 20 SNPs from the GWAS were localised in 11 genome regions. These regions contain 412 genes with human homologues, and functions could be assigned to 375 of the genes. A novel gene set analysis (GSA) was carried out to test the significance of the physics model key parameters, cell–cell adhesion, cytoskeleton organisation (cell stiffness) and blood flow, allowing the complex nature of the trait to be incorporated into the analysis through the grouping of genes by related functions. The most significant gene sets represented the functions of cytoskeleton organisation (cell stiffness) and blood flow, highlighting these as key processes in the pathology of EIPH. Biomarkers for hypertension were also found to be significant suggesting that genetic variation impacting blood pressure could also be important, perhaps as a consequence of the relationship between blood pressure and flow.

Several genes were contained within more than one gene set, with a sub-set of five genes affecting all three biological functions known to represent the key parameters in the physical model (cell–cell adhesion, cytoskeleton organisation and blood flow). These genes (*ABAT*, *CYFIP2*, *EMP2*, *FN1*, and *TEK*) are linked through their roles in promoting angiogenesis, particularly in response to hypoxic stress. TEK is the receptor for angiopoietin-1; the angiopoietin-1/TEK signalling complex regulates both maintenances of vascular quiescence and promotion of angiogenesis, with distinct signalling complexes being produced in the presence or absence of endothelial cell–cell adhesions [34]. Angiopoietin-1/TEK signalling also promotes microvascular integrity through interaction with the hypoxia-inducible factor HIF-2α. *CYFIP2* is a target gene for p53, a protein essential for cellular response to oxygen stress which is regulated by HIF-1 [35]. *EMP2* is upregulated under hypoxic conditions, promoting vascular endothelial growth factor (VEGF) expression through a HIF-1α dependent pathway [31]. *ABAT* is involved in response to hypoxia, and negative regulation of blood pressure (GO Biological Processes). The *FN1* gene codes for the type I fibrin-binding domain in fibronectin. Polymerized fibrin is important in the process of coagulation and wound healing; faulty assembly or premature lysis of fibrin would increase the risk of haemorrhage.

The hypoxia-inducible factors (HIFs), especially HIF-1α and HIF-2α, are key mediators of the adaptive response to hypoxic stress and play essential roles in maintaining lung homeostasis. Both human and animal genetic studies have shown that variants in HIF correlate with pulmonary vascular pathology and chronic lung diseases [36], including pulmonary arterial hypertension [37]. It has been postulated that inhibiting HIF could be a therapeutic target for pulmonary arterial hypertension (PAH) in humans [38]. Given the apparent similarities in the genetic background between PAH and EIPH, the inhibition of HIF might also provide therapeutic benefits for EIPH. Exercise and hypoxic conditions both increase angiogenesis, however, HIF signalling and gene expression are different under the two conditions. Exercise induces much larger changes in HIF expression levels compared with hypoxia alone [39]. Regular endurance training reduces skeletal muscle HIF expression under normoxic conditions [40], suggesting that HIF is involved in regulating adaptive gene response to exercise. Consequently, genetic variants increasing EIPH risk may also have effects on the HIF signalling pathway and response to training. Risk variants may have been maintained within the Thoroughbred racehorse population because they enhance muscle development and cardiovascular performance, as long as bleeding does not occur detrimentally.

The mechano-sensing roles of these genes are also important in vascular remodelling, as vascular endothelial cells are sensitive to stress and strain generated by blood flowing through the capillaries and venules. The mechano-sensitive and hypoxia responses are inter-linked; the hypoxia response has been shown to have a role in the differential expression of mechanosensitive genes within osteocytes [41], and it therefore seems plausible to expect that this relationship would also hold for other cells, such as vascular endothelial cells, which must respond to mechanical stresses. It is already known, for example, that microvascular integrity (vessel leakiness) is mediated by the role of angiopoietin 1 through a mechano-transduction pathway [42]. The interaction between the response to hypoxia and mechanical forces acting on endothelial cells appears to play a pivotal role in vascular remodelling, and the relationship between these responses merits further investigation. The nature of gene–gene interactions and pleiotropy within both the hypoxia and mechano-transduction pathways also need to be better understood before firm conclusions can be drawn on the suitability of therapeutic targets for preventing or reducing EIPH.

The physical model linking the EIPH phenotype to cellular biology and genetics has enabled candidate genes to be identified based on an understanding of their functional significance within the model. Further dissection of the biological pathways identified will elucidate the physiological benefits of genetic variants, in terms of cardiovascular enhancement and response to training/racing and the associated pathological risk of EIPH. This will enable improved therapies to be developed, as well as providing information required to reduce genetic risk within the Thoroughbred population.

## Figures and Tables

**Table 1:** Genome regions defined by the top 20 SNPs from the genome-wide association analysis (GWAS) for which gene lists were downloaded from Ensembl (www.ensembl.org).

Chromosome	Region (Base-Pair Position)
1	59,260,537–66,145,680
3	27,229,795–43,322,328
6	6,986,453
8	73,183,865–73,188,285
13	34,675,399–36,166,213
14	20,883,247–22,023,889
15	11,539,208 and 88,857,517
23	47,893,418
25	15,695,787–19,569,429
26	35,008,287
29	25,863,916

**Table 2:** Gene set analysis results for SNPs within gene boundaries.

Gene Set	No Genes	No SNPs	Chi-Square (Observed)	*p*-Value	Top SNP *p*-Value	Top SNP
Cell–cell adhesion	43	59	90.73	0.0625	0.00018	BIEC2-287067
Cytoskeleton	50	48	85.89	0.0267	0.00044	BIEC2-26621
Blood flow	26	61	114.95	0.0198	0.00025	BIEC2-1062654
Stroke	33	44	37.00	0.6322	0.0207	BIEC2-761299
Hypertension	29	54	97.96	0.0257	0.00044	BIEC2-26621
Random	258	121	177.12	0.0675	1.76 × 10^−6^	BIEC2-234438

**Table 3:** Gene set analysis results for SNPs within 5 kb of gene boundaries.

Gene Set	No Genes	No SNPs	Chi-Square (Observed)	*p*-Value	Top SNP *p*-Value	Top SNP
Cell–cell adhesion	43	64	95.25	0.0747	0.00019	BIEC2-287067
Cytoskeleton	50	53	99.22	0.0158	0.00044	BIEC2-26621
Blood flow	26	55	117.20	0.0066	0.00025	BIEC2-1062654
Stroke	33	47	38.42	0.6691	0.0207	BIEC2-761299
Hypertension	29	58	101.55	0.0309	0.0004	BIEC2-26621
Random	258	145	226.31	0.0378	1.76 × 10^−6^	BIEC2-234438

**Table 4:** Gene set analysis results for SNPs within 20 kb of gene boundaries.

Gene Set	No Genes	No SNPs	Chi-Square (Observed)	*p*-Value	Top SNP *p*-Value	Top SNP
Cell–cell adhesion	43	72	99.93	0.1053	0.00019	BIEC2-287067
Cytoskeleton	50	72	112.95	0.0473	0.00044	BIEC2-26621
Blood flow	26	63	132.51	0.0058	0.00025	BIEC2-1062654
Stroke	33	55	41.70	0.7610	0.0207	BIEC2-761299
Hypertension	29	68	114.64	0.0351	0.0004	BIEC2-26621
Random	258	195	287.99	0.0506	1.76 × 10^−6^	BIEC2-234438

**Table 5:** Haplotypes spanning the genes *ABAT* and *CYFIP2* which were significantly associated with the EIPH phenotype. At the *ABAT* gene, the haplotype GGGG was associated with increased risk, and the AGGA haplotype with reduced risk. A haplotype at the *CYFIP2* gene was associated with increased risk.

Chromosome	BP Position	Gene	Haplotype	β	*p*-Value
13	35,047,741–35,171,447	ABAT	GGGG	0.084	7.44 × 10^−8^
13	35,047,741–35,171,447	ABAT	AGGA	−0.037	0.013
14	21,768,636–22,023,889	CYFIP2	GAAAAAAGAGG	0.151	0.00025

**Figure A1:**
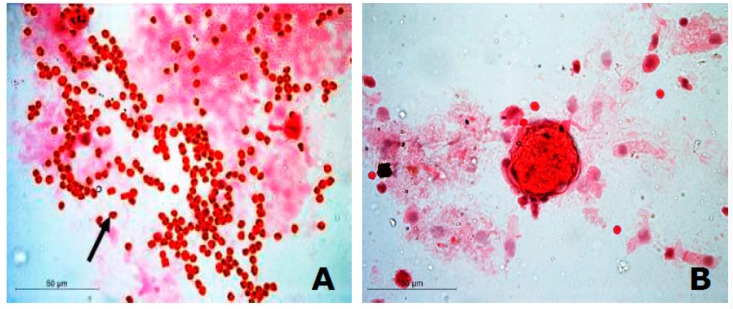
Cytology images depicting an equine tracheal wash sample scored as 3 for the presence of red blood cells (**A**) and haemosiderophages (**B**).

**Figure A2:**
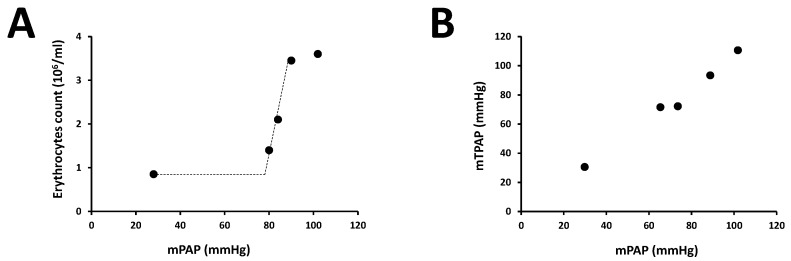
(**A**) Impact of the mean pulmonary arterial pressure (mPAP) on bleeding, data from [21]. (**B**) mPAP is not fully compensated by any other pressure and as a result, the mean transmural pulmonary arterial pressure (mTPAP) follows mPAP trends, data from [43].

**Figure A3:**
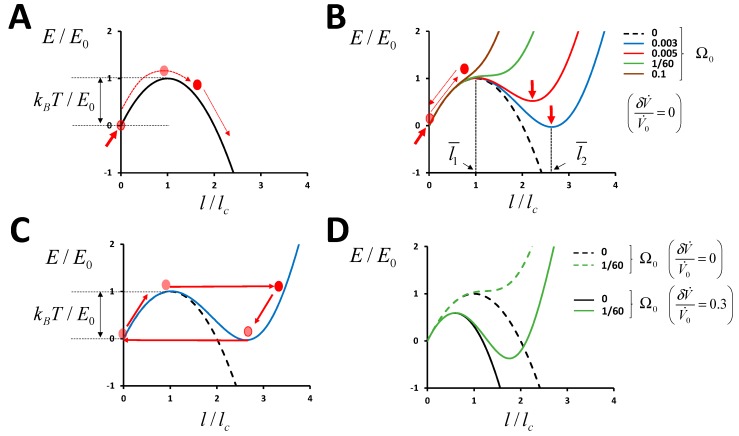
(**A**) Fracture definition in material physics: The initial state (thick red arrow) can be modulated by the thermal energy and when the tension is enough a fracture opens up in which case the red circle, which schematically represents the state of the fracture, ‘jumps’ above the energy barrier to generate fractures which are the largest possible. In this model there is no compensatory mechanism (i.e., a reduction of the tension due to fracture) hence the description ‘the largest possible’ remains valid. (**B**) When a compensatory mechanism is involved, namely when there is reduction of the tension due to the fracture, new stable states (e.g., thick red arrows on the right) emerge from the system leading to sustained fracture. However, the ability of the fracture to be sustained is a function of the value of omega which presents an inflection when its value is above 1/60 (green curve). In this case fractures do not appear however much the fracture can ‘jump’. It is the value of omega and thus the type of vessel that will determine whether the fracture is maintained (leading to a bleeding state) or not. The maximum and minimum value of the energy are located at ${\bar{l}}_{1}$ and ${\bar{l}}_{2}$, respectively. (**C**) An interesting phenomenon is the bleeding oscillation that should occur when the tension energy is sufficiently large for the system’s energy to match the thermal energy. In this context constant opening and closing configurations are possible. (**D**) In the case of increased bloodflow, the system’s energy landscape changes leading to the appearance of new stable modes (i.e., fracture lengths) that were not present before.

## Supplementary Materials

The following are available online at https://www.mdpi.com/2073-4425/10/11/880/s1, Table S1: Gene ontology terms, Table S2: SNP based estimates of genetic variance, Table S3: EIPH Top 20 SNPs gene functions, Table S4: Gene set lists.

## Appendix B

### Appendix B.1. A Physics-Based Model of EIPH

Physiological studies have shown that during intense exercise a mean pulmonary arterial pressure (mPAP) above 80 mmHg is required to elicit bleeding in the lungs (Figure A1A) [21]. Such an increase in mPAP is not fully compensated by any other pressure and as a result the mean transmural pulmonary arterial pressure (mTPAP) follows mPAP trends (Figure A1B) [43] and the pressure across the capillary wall (mTPAP) is expected to promote a large increase in the capillary wall stress estimated to be $\sim 8.3\times 10^{4}\;{N/m}^{2}$ for an mTPAP ~95 mmHg and capillary radius ~3.3 μm during exercise [2]. Using the surface tension $\sim h\times 8.3\times 10^{4}\;{N/m}^{2}$ where h~10 μm is the capillary wall thickness [44] one finds $\sim 8.3\times 10^{-1}\;N/m$. The physics model leads to two important conclusions: the first is that bleeding occurs under the form of an abrupt transition, which means that standard physics theory relating to a critical phenomenon (or phase transitions) should apply. The second is that the most important variable is the intra-capillary pressure which is not compensated and is linked to blood flow. In other words, the mTPAP written as $P_{i}-P_{e}$ where the indexes ‘i’ and ‘e’ refer to intra- and extra- respectively, can be rewritten as, $P_{i}-P_{e}^{0}$.

Modelling the pathophysiology of blood vessel rupture requires modification of the physical model to incorporate biology. We have decomposed the problem into smaller steps which, when resolved and integrated, provide a better understanding of the pathophysiology of EIPH. The first step corresponds to determining the physical conditions for a vessel to rupture provided that the vessel has a constant lumen radius. The second step is to add to this the risk of bleeding and in particular the continuation of bleeding when the horse is exercising. The third step is to consider the contractile ability of vessels (i.e., arterioles or venuoles) to modulate vascular blood flow and pressure, and the relationship with bleeding. Once integrated these steps show the sensitivity of EIPH to key physical parameters.

#### Appendix B.1.1. Part 1—Physics of Blood Vessel Rupture with Constant Radius under an Imposed Hydrostatic Pressure

Physics theory can be applied to any vessel provided that their radius is constant. The first point to thus resolve is, given the fragility of capillaries, what capillary hydrostatic pressure is needed to rupture them in such a way that the rupture is large enough to let blood cells flow out. To model this phenomenon, we make use of the theory developed in physics to understand how fracture occurs in materials. We assume that capillaries can be modelled as a small shallow cylinder with a wall thickness h constant length $L_{0}$ and radius R. In this context, the entire volume of the capillary is $V=\pi L_{0}R$ with a surface area of $S=2\pi L_{0}R$. The mechanical tension or surface tension with unit $J/m^{2}$ that is applied to the capillary wall is noted as, $h\sigma_{0}$, where $\sigma_{0}$ is the stress that is present within the capillary wall, which is assumed to be independent of the wall thickness. Laplace’s law then gives the relationship between the radius of the capillary, the surface tension and the pressure difference between the lumen of the capillary and the alveoli: $h\sigma_{0}\sim R\left(P_{i}-P_{e}\right)$. This last relation states that as the pressure difference increases, the surface tension increases in a similar proportion and it is expected that the increase in surface tension will fracture the capillary.

To model the possibility of a capillary fracture of surface area ‘s’ it is noted that any fracture releases an energy $\sim -sh\sigma_{0}^{2}/E$ where, $\sigma_{0}^{2}/E$, is the energy density and E the elastic modulus of the capillary wall and, sh is the volume generated by the fracture. The fracture can only appear if cell–cell de-adhesion occurs so that cells can move apart. Assuming that the density of cell–cell adhesion molecules is constant along the contour of cells, then the energy is proportional to the contour length of the fracture (the fracture ‘lips’). Noting by the variable ‘l’ the contour length and by ‘hλ’ the cell–cell binding energy per unit of contour (i.e., λ is an energy per surface area of adhesion), a drop in the surface tension energy is always accompanied by an increase of energy due to the presence of free lips given by ~2λhl where the factor ‘2′ is added as each time a fracture appears two lips are generated. Let us consider that the contour length is proportional to the fracture area under the form: $s\sim al^{2}$ where a is a geometrical constant such that for a circular fracture, a=1/4π. The variation of surface energy can be written as, $E_{S}\sim 2\lambda hl-al^{2}h\sigma_{0}^{2}/E$. The surface energy represented in Figure A2(A) demonstrates that not all fractures diverge irreversibly but that a critical fracture size, $l_{c}\sim E\lambda /a\sigma_{0}^{2}$, needs to be attained. This allows $E_{S}\sim E_{0}\left(2\bar{l}-{\bar{l}}^{2}\right)$ to be rewritten with $E_{0}\sim hE\lambda^{2}/a\sigma_{0}^{2}$ and $\bar{l}=l/l_{c}$ (Figure A3(A)). This means that the capillaries can heal physically (and biologically) provided that the pressure difference or surface tension is small enough. This has been demonstrated in cell culture [45].

#### Appendix B.1.2. Part 2—Physics of Blood Vessel Rupture with the Presence of a Leak under an Imposed Hydrostatic Pressure

The theory applied from material physics suggests that fractures diverge once a threshold size, $l_{c}\sim E\lambda /a\sigma_{0}^{2}$, has been attained. However, in the vascular system, it is the blood flow that builds up the pressure inside vessels and if a leak occurs then it is possible to demonstrate that a fracture or rupture can be maintained. This point is important as racehorses undergoing intense exercise for a given time will probably bleed during the time considered. As shown above, the surface tension linked to the pressure inside vessels is also affected by the blood leaking as it is expected to lower the transmural capillary pressure. To model this we consider the internal volume of a capillary. The pressure difference changes are related to the amount of blood flowing into the capillary section under consideration. The extra blood flow rate in the capillary is noted as ‘$\delta \dot{V}$’ and ‘τ’ is the characteristic time related to the length of time the flow rate is increased. Note that the characteristic time is supposed to be long enough to apply thermodynamics and $\delta \dot{V}$ can be related to basic haemodynamics for steady-state flows, i.e., the Hagen–Poiseuille equation. The product $\delta \dot{V}\tau$ is therefore the extra volume present in the capillary and it is natural to assume that $\delta \dot{V}\tau$ should increase the value of the lumen pressure, $P_{i}$. To link $P_{i}$ and $\delta \dot{V}\tau$ a simple model is used whereby the volume of the capillary under consideration is constant. If more blood is poured into the section, $P_{i}$ is expected to increase as a function of the proportion of extra volume being poured in. That is to say that for an incompressible fluid, i.e., the blood, at the lowest order $P_{i}\sim P_{i}^{0}\left(1+\delta \dot{V}\tau /V\right)$ is obtained where $P_{i}^{0}$ is the lumen pressure under normal/resting conditions. This relation allows the pressure difference to be written as, $P_{i}-P_{e}\sim \left(P_{i}^{0}-P_{e}\right)+P_{i}^{0}\delta \dot{V}\tau /V$. However, a fraction of this extra volume can be leaked out from the capillary lumen. Considering a capillary fracture of surface area ‘s’, the volume leaked per unit of time is therefore ~s×v where ‘v’ is the blood velocity leaking out. Using Darcy’s law, the velocity is a function of the pressure difference between the inside and outside of the vessel, blood viscosity ‘μ’ and capillary wall thickness ‘h’, expressed under the form, $v\sim \alpha \left(P_{i}-P_{e}\right)s/\mu h$, where ‘α’ is the proportionality constant. Using $s\sim al^{2}$, the balance of volumes, i.e., what flows in minus what leaks out, can be re-expressed as: $\delta \dot{V}\tau -a^{2}\alpha \tau \left(P_{i}-P_{e}\right)l^{4}/\mu h$. As a result the pressure difference in the presence of blood leaking out from the vessel can be rewritten as, $P_{i}-P_{e}\sim P_{i}^{0}-P_{e}+P_{i}^{0}\left(\delta \dot{V}\tau /V-a^{2}\alpha \tau \left(P_{i}-P_{e}\right)l^{4}/V\mu h\right)$, which once rearranged gives: $P_{i}-P_{e}\sim \left(P_{i}^{0}-P_{e}+P_{i}^{0}\delta \dot{V}\tau /V\right)/\left(1+a^{2}\alpha \tau P_{i}^{0}l^{4}/V\mu h\right)$. It follows by virtue of Laplace’s law that new surface tension, $h\sigma \sim R\left(P_{i}-P_{e}\right)$, can be defined: $h\sigma \sim R\left(P_{i}^{0}-P_{e}+P_{i}^{0}\delta \dot{V}\tau /V\right)/\left(1+a^{2}\alpha \tau P_{i}^{0}l^{4}/V\mu h\right)$. Noting $h\sigma_{0}\sim R\left(P_{i}^{0}-P_{e}\right)$ and $\tau RP_{i}^{0}/Vh\sigma_{0}=1/{\dot{V}}_{0}$ one obtains: $\sigma \sim \sigma_{0}\left(1+\delta \dot{V}/{\dot{V}}_{0}\right)/\left(1+a^{2}\alpha \tau P_{i}^{0}l^{4}/V\mu h\right)$. In the expression of the new surface tension the presence of ‘$l^{4}$’ in the denominator confirms that the surface tension of the capillary decreases as a function of the rupture length. Within the latter relation the characteristic time ‘τ’ remains present but as a steady-state flow is considered it can be related to usual hemodynamic parameters. A simple dimensional analysis allows one to find that surface tension can also be expressed as a function of the steady-state flow rate in resting conditions under the form $h\sigma_{0}\sim {\dot{V}}_{0}\mu L_{0}/R^{3}$. As $\tau RP_{i}^{0}/Vh\sigma_{0}=1/{\dot{V}}_{0}$, by identity one obtains, $\tau P_{i}^{0}/\mu \sim {\left(L_{0}/R\right)}^{2}$. Reintegrating these relations into the system’s energy one finds:

(A1) $$\begin{array}{l} E_{S}/E_{0}\sim 2\bar{l}-{\bar{l}}^{2}{\left(\frac{1+\delta \dot{V}/{\dot{V}}_{0}}{1+\Omega_{0}{\bar{l}}^{4}}\right)}^{2}\\ \Omega_{0}=a^{2}\alpha {\left(L_{0}/R\right)}^{2}l_{c}^{4}/Vh\\ l_{c}\sim E\lambda /a\sigma_{0}^{2} \end{array}$$

Equation (A1) plotted in Figure A3(B) captures the physical elements that are deemed important for EIPH. The bleeding transition can now be considered.

#### Appendix B.1.3. Part 3—Physical Conditions for the Bleeding Transition

While Equation (A1) captures most of the physics of EIPH, under this form it cannot provide any meaningful relationship between the physical variables supposedly involved in EIPH. However, it is clear that capillaries with poor cell–cell adhesion will probably have a tendency to bleed more, a result that comes automatically from Equation (A1). As a result, another relation needs to be introduced that links the physical variables together to determine the ‘bleeding transition’.

The ‘no-bleeding’ condition up to the fracture can be captured by setting $\Omega_{0}\sim 0$ (this is the equivalent of saying that α~0 in the absence of leaks), which transforms Equation (A1) into $E_{S}/E_{0}\sim 2\bar{l}-{\bar{l}}^{2}{\left(1+\delta \dot{V}/{\dot{V}}_{0}\right)}^{2}$. It is clear that the latter relation has an extreme value that is modulated by the blood flow and that is given when ‘${\bar{l}}_{1}$’ the solution of: $\partial \left(E_{S}/E_{0}\right)/\partial \bar{l}\sim 0$ leading to ${\bar{l}}_{1}\sim 1/{\left(1+\delta \dot{V}/{\dot{V}}_{0}\right)}^{2}$ with a resulting extreme value for the energy: ${\left(E_{S}\right)}_{\max}\sim E_{0}/2{\left(1+\delta \dot{V}/{\dot{V}}_{0}\right)}^{2}$. The blood flow, therefore, modulates the energy barrier leading to rupture. Furthermore the thermal energy $k_{B}T$ (where $k_{B}$ is Boltzmann’s constant and T the absolute temperature) modulates the energy of this fundamental state to generate statistical thermal fluctuations and it is when this fluctuation is enough to promote a fracture that the bleeding transition occurs. Putting ${\left(E_{S}\right)}_{\max}\sim k_{B}T$, i.e., that the thermal energy needs to match the extrema value of the potential, one finds a simple critical relation:

(A2) $$2{\left(1+\delta {\dot{V}}_{c}/{\dot{V}}_{0}\right)}^{2}k_{B}T\sim hE\lambda^{2}/a\sigma_{0}^{2}\;,$$

where $\delta {\dot{V}}_{c}/{\dot{V}}_{0}={\dot{V}}_{c}/{\dot{V}}_{0}-1$ and ${\dot{V}}_{c}$ correspond to a critical flow rate that using Equation (A2) can be rewritten simply as: ${\dot{V}}_{c}\sim {\dot{V}}_{0}\sqrt{hE\lambda^{2}/2a\sigma_{0}^{2}k_{B}T}$. Using $l_{c}\sim E\lambda /a\sigma_{0}^{2}$ (Equation (A1)) one obtains a simple expression of ${\dot{V}}_{c}$ under the form: ${\dot{V}}_{c}\sim {\dot{V}}_{0}\sqrt{hl_{c}\lambda /2k_{B}T}$.

As the model is expected to provide prior-information to genetic analysis it is essential to demonstrate its coherence with existing data. The relation, ${\dot{V}}_{c}\sim {\dot{V}}_{0}\sqrt{hE\lambda^{2}/2a\sigma_{0}^{2}k_{B}T}$, derived from Equation (A2) links the macroscopic parameter, ${\dot{V}}_{c}/{\dot{V}}_{0}$, to microscopic ones, $hE\lambda^{2}/2a\sigma_{0}^{2}\;k_{B}T$, that have all been separately determined experimentally. Given that capillary rupture occurs at the level of cell–cell adhesion, as the interaction energy involving cell–cell adhesion complexes is typically $\sim 100\;k_{B}T$ [46] and that the surface density for adhesive units involved between adjacent cells $\sim 10^{3}\;{\mu m}^{-2}$ [47], one deduces a typical value of $\lambda \sim 10^{5}\;k_{B}{T/\mu m}^{2}$. Using $k_{B}T\sim 3\times 10^{-21}J$ (at T~300 K i.e., a temperature of ${27\;}^{o}C$) leads to $\lambda \sim 3\cdot 10^{-4}\;J/m^{2}$. Considering capillary stress $\sigma_{0}\sim 8\times 10^{4}\;N/m^{2}$ [3], an elastic modulus per cell $E\sim 10^{3}\;Pa$ [48], and a typical capillary wall thickness $h\sim 10^{-6}\;m$ and a=1/4π one finds $\sqrt{hE\lambda^{2}/2a\sigma_{0}^{2}k_{B}T}\sim 5-6$. To determine ${\dot{V}}_{c}/{\dot{V}}_{0}$ it can be legitimately assumed that $\delta {\dot{V}}_{c}/{\dot{V}}_{0}$ is linearly linked to the transmural pressure variation inside the vessel (This assumption can be justified as follow: Using $P_{i}-P_{e}\sim \left(P_{i}^{0}-P_{e}\right)+P_{i}^{0}\delta \dot{V}\tau /V$, the blood flow can be related to a critical pressure difference, i.e., a critical transmural pressure, under the form: $\delta P_{c}\sim P_{i}^{0}\delta \dot{V}\tau /V\sim P_{i}^{0}\times \left({\dot{V}}_{c}/{\dot{V}}_{0}-1\right)$.) namely, $\delta P_{c}/P_{0}\sim \delta {\dot{V}}_{c}/{\dot{V}}_{0}$. Then the use of Figure A2(A) allows one to predict that ${\dot{V}}_{c}/{\dot{V}}_{0}\sim 4$ (or $\delta {\dot{V}}_{c}/{\dot{V}}_{0}\sim 3$). The order of magnitudes is similar, suggesting that the model is sound to be used further.

Based on the similarity above, one can also provide an order of magnitude for $l_{c}$ that can be rewritten as, $l_{c}/h\sim \lambda \times E/ah\sigma_{0}^{2}$. As described above, the term $\sigma_{0}^{2}/E$ is the energy density of the capillary wall meaning that, $h\sigma_{0}^{2}/E$, has an order of magnitude similar to the surface tension of the wall. Using $h\sigma_{0}^{2}/E\sim 0.8\;N/m$ and a=1/4π one finds $l_{c}\sim 30\;\mathrm{nm}$. It is worth noting that with the value of $l_{c}$ now defined, it is expected that $\Omega_{0}<<1$ as $\Omega_{0}\sim l_{c}^{4}$. While Equation (A2) provides the physical conditions required to be fulfilled for bleeding it does not provide any information on the amount of blood crossing the vessel wall.

#### Appendix B.1.4. Part 4—Physical Pathophysiology of EIPH

The main question is to find the relation that allows sustained bleeding to occur. Considering $\Omega_{0}<<1$, Equation (A1) can be rewritten as a Taylor’s expansion (chain rule method) up to the 6th power of the energy around $\bar{l}\sim 0$ under the form:

(A3) $$E_{S}/E_{0}\sim 2\bar{l}-{\left(1+\delta \dot{V}/{\dot{V}}_{0}\right)}^{2}{\bar{l}}^{2}+2\Omega_{0}{\left(1+\delta \dot{V}/{\dot{V}}_{0}\right)}^{2}{\bar{l}}^{6}.$$

The term in ‘${\bar{l}}^{6}$’ provides for maintenance of the leak. As shown in Figure A3(B), Equation (A3) demonstrates the presence of two extrema. The order of magnitude of the maximum, noted ${\bar{l}}_{1}$, results from the competition between the two first terms that are present in the right-hand-side of Equation (A3) leading to ${\bar{l}}_{1}\sim 1/{\left(1+\delta \dot{V}/{\dot{V}}_{0}\right)}^{2}$, which is one solution determined previously. Likewise the minimum, noted ${\bar{l}}_{2}$, results from the competition between the two last terms that are present in the right-hand-side of Equation (A3), leading to ${\bar{l}}_{2}^{4}\sim 1/\Omega_{0}$. It is worth noting here that using Equation (A1) to replace $\Omega_{0}$ by its literal expression one finds, $l_{2}^{}\sim R{\left(\pi h/a^{2}\alpha L_{0}\right)}^{1/4}$, that is to say that the size of the capillary rupture is chiefly imposed by the radius of the capillaries. Finally, the maintenance of the leak appears only if ${\bar{l}}_{2}\geq {\bar{l}}_{1}$ that is: $\left(R/l_{c}\right){\left(\pi h/a^{2}\alpha L_{0}\right)}^{1/4}\geq 1/{\left(1+\delta \dot{V}/{\dot{V}}_{0}\right)}^{2}$. In the latter relation there are only two meaningful physical and physiological parameters, namely $l_{c}$ and $\delta \dot{V}/{\dot{V}}_{0}$ that, as a result, suggests that the leak can be maintained only if the ratio $l_{c}/{\left(1+\delta \dot{V}/{\dot{V}}_{0}\right)}^{2}$ is smaller than a given constant, i.e., $R{\left(\pi h/a^{2}\alpha L_{0}\right)}^{1/4}$, which is linked to the geometry of the capillaries. In view of this last result the prior information that physics gives in terms of genetic sensitivity to EIPH can be discussed. To start with the critical length, $l_{c}\sim E\lambda /a\sigma_{0}^{2}$, is a ratio between the energy involved in cell–cell adhesion and the stiffness of cells suggesting that if the adhesion diminishes, for example by a reduction in the number of cadherins, and/or that the stiffness of cells increases, for example by augmenting the stiffness of the cytoskeleton, EIPH is more likely to occur. However the presence of the squared term in ratio, $l_{c}/{\left(1+\delta \dot{V}/{\dot{V}}_{0}\right)}^{2}$, means that EIPH is potentially more sensitive to the blood flowing into capillaries which may result from physiological or anatomical changes in the vascular system, for example when venules have a smaller diameter [6,7,8] or following the selection of racehorses with larger cardiac outputs [49]. In any case, contrary to what has previously been suggested [50], blood viscosity does not seem to be involved.

## Appendix C

### Genome-Wide Association Top 20 SNPs

**Table A1:** Top 20 SNPs from the GWAS with unadjusted (Unadj), Bonferroni single step adjusted (Bonf), Benjamini and Hochberg (1995) step-up false discovery rate control (FDR_BH) and Benjamini and Yekutieli (2001) step-up false discovery rate control (FDR_BY) *p-*values.

Chromosome	SNP Name	Unadj	Bonf	FDR_BH	FDR_BY
3	BIEC2-774953	1.25 × 10^−7^	0.0055	0.0055	0.0613
23	BIEC2-626135	4.48 × 10^−7^	0.0195	0.0097	0.1095
13	BIEC2-233640	1.06 × 10^−6^	0.0450	0.0153	0.1718
13	BIEC2-233687	1.76 × 10^−6^	0.0763	0.0153	0.1718
13	BIEC2-234438	1.76 × 10^−6^	0.0763	0.0153	0.1718
13	BIEC2-233294	2.11 × 10^−6^	0.0919	0.0153	0.1724
1	BIEC2-27502	1.83 × 10^−5^	0.7942	0.1135	1
13	BIEC2-233191	4.50 × 10^−5^	1	0.2445	1
25	BIEC2-662769	9.31 × 10^−5^	1	0.4497	1
14	BIEC2-248255	0.000117	1	0.4641	1
14	BIEC2-248094	0.000117	1	0.4641	1
6	BIEC2-938252	0.000162	1	0.5746	1
15	BIEC2-325838	0.000182	1	0.5746	1
15	BIEC2-287067	0.000185	1	0.5746	1
26	BIEC2-693883	0.000253	1	0.5763	1
8	BIEC2-1062654	0.000253	1	0.5763	1
8	BIEC2-1062668	0.000253	1	0.5763	1
25	BIEC2-661835	0.000257	1	0.5763	1
29	BIEC2-760805	0.000268	1	0.5763	1
1	BIEC2-24798	0.000364	1	0.5763	1

## Appendix D

### Epistatic Interactions between SNPs in Five Key Genes from the Gene Set Analysis with Biological Functions Relating to Cell–cell Adhesion, Cytoskeleton Organisation (Cell Stiffness) and Blood Flow

**Table A2:** Significant epistatic interactions (*p* < 0.05) between SNPs on chromosomes 6, 13, 14 and 23 located within or flanking the genes *ABAT*, *EMP2*, *CYFIP2*, *FN1* and *TEK*.

Chr 1	SNP 1	Chr 2	SNP 2	β Interaction	*p*-Value
6	BIEC2-937111	14	BIEC2-248213	0.068	0.046
6	BIEC2-937111	23	BIEC2-625265	0.062	0.026
13	BIEC2-232441	14	BIEC2-248202	−0.042	0.036
13	BIEC2-232441	14	BIEC2-248209	−0.043	0.018
13	BIEC2-232441	14	BIEC2-248210	−0.056	0.001
13	BIEC2-232441	14	BIEC2-248213	0.060	0.001
13	BIEC2-232441	14	BIEC2-248227	0.069	0.000
13	BIEC2-232441	14	BIEC2-248229	0.070	0.000
13	BIEC2-232441	14	BIEC2-248255	0.202	0.012
13	BIEC2-232441	23	BIEC2-625253	−0.060	0.010
13	BIEC2-232525	14	BIEC2-248202	−0.067	0.021
13	BIEC2-232525	14	BIEC2-248213	0.058	0.027
13	BIEC2-232525	14	BIEC2-248227	0.065	0.016
13	BIEC2-232525	14	BIEC2-248229	0.055	0.038
13	BIEC2-233585	13	BIEC2-233626	−0.063	0.007
13	BIEC2-233585	14	BIEC2-248202	−0.050	0.012
13	BIEC2-233585	14	BIEC2-248227	0.035	0.044
13	BIEC2-233585	14	BIEC2-248229	0.035	0.043
14	BIEC2-248202	14	BIEC2-248210	0.038	0.013
14	BIEC2-248202	14	BIEC2-248213	−0.036	0.030
14	BIEC2-248202	14	BIEC2-248227	−0.047	0.018
14	BIEC2-248202	14	BIEC2-248229	−0.040	0.019
14	BIEC2-248209	14	BIEC2-248227	−0.042	0.049
14	BIEC2-248210	14	BIEC2-248213	−0.039	0.039
14	BIEC2-248210	14	BIEC2-248227	−0.045	0.014
14	BIEC2-248210	14	BIEC2-248229	−0.043	0.019
14	BIEC2-248213	14	BIEC2-248227	0.043	0.012
14	BIEC2-248213	14	BIEC2-248229	0.041	0.017
14	BIEC2-248227	14	BIEC2-248229	0.041	0.017
14	BIEC2-248255	23	BIEC2-625247	−0.106	0.026
14	BIEC2-248255	23	BIEC2-625253	−0.201	0.012
14	BIEC2-248255	23	BIEC2-625265	−0.098	0.039
