# Supplementary material for: A Mechanogenetic Model of Exercise-Induced Pulmonary Haemorrhage in the Thoroughbred Horse
Source: Genes (Basel). 2019 Nov 1;10(11):880. doi: 10.3390/genes10110880 (PMC6895809; doi:10.3390/genes10110880)
Supplement: Supplementary file 1 [file genes-10-00880-s001.zip › Blott et al Supplementary Files/Supplementary File S1 Gene Ontology Terms.docx]

**Table S1**. Gene Ontology (GO) terms used to identify gene sets for the three key model parameters

| **Cell-cell adhesion** |
| --- |
|  |
| Regulation of cell-cell adhesion |
| 🡪 regulation of cell adhesion |
| Positive regulation of cell-cell adhesion |
| 🡪 positive regulation of cell adhesion |
|  |
| Cell-cell junction assembly |
| 🡪 cell junction assembly |
| 🡪🡪 cell junction organisation |
| Cell-cell junction organisation |

| **Blood flow** |
| --- |
|  |
| Blood pressure/systemic arterial blood pressure |
| Blood circulation |
| Blood volume (including renal system process involved in regulation of above terms) |
| Blood vessel remodelling |
| Vasoconstriction |
| 🡪 negative regulation of blood vessel diameter |
| 🡪 regulation of blood vessel diameter |
| 🡪 regulation of tube diameter |
| 🡪 regulation of tube size |
| 🡪 regulation of blood vessel size |
| 🡪🡪 vascular process in circulatory system |
| Artery morphogenesis |
| Artery development |
| Blood vessel development |
| Vasculature development |
| Blood vessel morphogenesis |
| Lung vasculature development |

| **Cytoskeleton organisation (cell stiffness)** |
| --- |
|  |
| Actin filament bundle assembly |
| Actin filament bundle organisation |
| Actin filament organisation |
| Cytoskeleton organisation |
| Actin filament-based process |
