# Supplementary material for: A Mechanogenetic Model of Exercise-Induced Pulmonary Haemorrhage in the Thoroughbred Horse
Source: Genes (Basel). 2019 Nov 1;10(11):880. doi: 10.3390/genes10110880 (PMC6895809; doi:10.3390/genes10110880)
Supplement: Supplementary file 1 [file genes-10-00880-s001.zip › Blott et al Supplementary Files/Supplementary File S2 SNP based Estimates of Genetic Variance.docx]

**Table S2.** SNP-based estimates of genetic variance for individual chromosomes

| **Chromosome** | **Genetic variance (heritability)** | **Standard error (SE)** |
| --- | --- | --- |
| 1 | 0.248 | 0.24 |
| 2 | 0.106 | 0.16 |
| 3 | 0.102 | 0.18 |
| 4 | 0.000 | 0.12 |
| 5 | 0.183 | 0.20 |
| 6 | 0.044 | 0.14 |
| 7 | 0.133 | 0.23 |
| 8 | 0.000 | 0.15 |
| 9 | 0.000 | 0.15 |
| 10 | 0.060 | 0.15 |
| 11 | 0.089 | 0.15 |
| 12 | 0.000 | 0.17 |
| 13 | 0.278 | 0.15 |
| 14 | 0.000 | 0.16 |
| 15 | 0.059 | 0.21 |
| 16 | 0.000 | 0.18 |
| 17 | 0.135 | 0.16 |
| 18 | 0.000 | 0.21 |
| 19 | 0.437 | 0.23 |
| 20 | 0.315 | 0.21 |
| 21 | 0.066 | 0.16 |
| 22 | 0.000 | 0.16 |
| 23 | 0.000 | 0.14 |
| 24 | 0.000 | 0.20 |
| 25 | 0.047 | 0.12 |
| 26 | 0.000 | 0.17 |
| 27 | 0.000 | 0.14 |
| 28 | 0.000 | 0.15 |
| 29 | 0.043 | 0.16 |
| 30 | 0.000 | 0.10 |
| 31 | 0.046 | 0.16 |
