# Supplementary material for: A Mechanogenetic Model of Exercise-Induced Pulmonary Haemorrhage in the Thoroughbred Horse
Source: Genes (Basel). 2019 Nov 1;10(11):880. doi: 10.3390/genes10110880 (PMC6895809; doi:10.3390/genes10110880)
Supplement: Supplementary file 1 [file genes-10-00880-s001.zip › Blott et al Supplementary Files/Supplementary File S4 Gene Set Lists.docx]

**Table S4**. Gene set lists

**Cytoskeleton Cell-Cell Adhesion Intersection**

| **Gene symbol** | **Gene name** |
| --- | --- |
| **ABAT** | **4 aminobutyrate aminotransferase** |
| ARPC2 | actin related protein 2/3 complex subunit 2 |
| CHMP5 | charged multivesicular body protein 5 |
| **CYFIP2** | **cytoplasmic FMR1-interacting protein 2** |
| **EMP2** | **epithelial membrane protein 2** |
| **FN1** | **fibronectin** |
| SOCS1 | suppressor of cytokine signalling 1 |
| **TEK** | **angiopoietin-1 receptor** |
| TNS1 | tensin 1 |
| VCL | vinculin |
| WHRN | whirlin |

**Cytoskeleton Blood Flow Intersection**

| **Gene symbol** | **Gene name** |
| --- | --- |
| AAMP | angio associated migratory cell protein |
| **ABAT** | **4 aminobutyrate aminotransferase** |
| **CYFIP2** | **cytoplasmic FMR1-interacting protein 2** |
| **EMP2** | **epithelial membrane protein 2** |
| **FN1** | **fibronectin** |
| KCNMA1 | potassium calcium-activated channel subfamily M alpha 1 |
| RAPIGDS1 | rap1 GTPase-GDP dissociation stimulator 1 |
| SGCD | sarcoglycan delta |
| **TEK** | **angiopoietin-1 receptor** |

**Cytoskeleton Hypertension Intersection**

| **Gene symbol** | **Gene name** |
| --- | --- |
| KCNAM1 | potassium calcium-activated channel subfamily M alpha 1 |
| SGCD | sarcoglycan delta |
| WHRN | whirlin |

**Cell-Cell Adhesion Blood Flow Intersection**

| **Gene symbol** | **Gene name** |
| --- | --- |
| **ABAT** | **4 aminobutyrate aminotransferase** |
| ADGRE2 | adhesion G protein-coupled receptor E2 |
| B4GALT1 | beta-1,4-galactosyltransferase 1 |
| CDH13 | cadherin 13 |
| **CYFIP2** | **cytoplasmic FMR1-interacting protein 2** |
| DLG5 | discs large MAGUK scaffold protein 5 |
| EMCN | endomucin |
| **EMP2** | **epithelial membrane protein 2** |
| **FN1** | **fibronectin** |
| FOXF1 | forkhead box 1 |
| IL12B | interleukin 12B |
| PLAU | plasminogen activator, urokinase |
| PPP3CA | protein phosphatase 3 catalytic subunit alpha |
| **TEK** | **angiopoietin-1 receptor** |

**Cell-Cell Adhesion Hypertension Intersection**

| **Gene symbol** | **Gene name** |
| --- | --- |
| CDH13 | cadherin 13 |
| CDH15 | cadherin 15 |
| FOXF1 | forkhead box 1 |
| GATA3 | gata binding protein 3 |
| IL12B | interleukin 12B |
| PRKCQ | protein kinase C theta |
| WHRN | whirlin |

**Blood Flow Hypertension Intersection**

| **Gene symbol** | **Gene name** |
| --- | --- |
| ADH1B | alcohol dehydrogenase 1B (class I), beta polypeptide |
| ADRA1B | adrenoceptor alpha 1B |
| CDH3 | cadherin 3 |
| CDH13 | cadherin 13 |
| CYBA | cytochrome B-245 alpha chain |
| FOXF1 | forkhead box 1 |
| IL12B | interleukin 12B |
| KCNMA1 | potassium calcium-activated channel subfamily M alpha 1 |
| SGCD | sarcoglycan delta |
| ZNF618 | zinc finger protein 618 |

**Cell-Cell Adhesion**

| **Gene symbol** | **Chromosome** | **Gene start (bp position)** | **Gene end (bp position)** |
| --- | --- | --- | --- |
| CDH23 | 1 | 59492523 | 59785104 |
| SPOCK2 | 1 | 59970116 | 59992878 |
| PLAU | 1 | 61551733 | 61556480 |
| VCL | 1 | 61615198 | 61710132 |
| DLG5 | 1 | 65017459 | 65144609 |
| ZMIZ1 | 1 | 66286005 | 66392640 |
| CDH13 | 3 | 30386191 | 31158287 |
| FOXF1 | 3 | 33360886 | 33363420 |
| PIEZO1 | 3 | 35232776 | 35252686 |
| CDH15 | 3 | 35656487 | 35667633 |
| SPG7 | 3 | 35931713 | 35963940 |
| ADGRE2 | 3 | 36485943 | 36493029 |
| PPP3CA | 3 | 38531893 | 38677807 |
| EMCN | 3 | 39159431 | 39170331 |
| ATIC | 6 | 5446142 | 5472875 |
| FN1 | 6 | 5486218 | 5551290 |
| IGFBP2 | 6 | 6594703 | 6598245 |
| TNS1 | 6 | 7610232 | 7705182 |
| ARPC2 | 6 | 7918353 | 7949094 |
| GPBAR1 | 6 | 7955751 | 7956835 |
| SOCS1 | 13 | 33226799 | 33227469 |
| EMP2 | 13 | 33776335 | 33785814 |
| ABAT | 13 | 35109975 | 35145460 |
| IL12B | 14 | 20194970 | 20203472 |
| CLINT1 | 14 | 21505357 | 21533756 |
| ADAM19 | 14 | 21691499 | 21767122 |
| CYFIP2 | 14 | 21808772 | 21911520 |
| HAVCR2 | 14 | 22058284 | 22072297 |
| LARP1 | 14 | 23945344 | 24022973 |
| ZAP70 | 15 | 11529127 | 11540466 |
| TEK | 23 | 44907561 | 45009946 |
| CHMP5 | 23 | 49566805 | 49712668 |
| B4GALT1 | 23 | 49574813 | 49594213 |
| UBAP2 | 23 | 49987217 | 50069809 |
| SVEP1 | 25 | 15545761 | 15711670 |
| AMBP | 25 | 18881436 | 18894743 |
| WHRN | 25 | 19220773 | 19305811 |
| TNC | 25 | 19705467 | 19766034 |
| IGSF5 | 26 | 35670473 | 35700218 |
| DSCAM | 26 | 35885736 | 36168133 |
| GATA3 | 29 | 26022567 | 26039578 |
| PRKCQ | 29 | 27411915 | 27488344 |
| IL2RA | 29 | 27893321 | 27899959 |

**Cytoskeleton organisation (cell stiffness)**

| **Gene symbol** | **Chromosome** | **Gene start (bp position)** | **Gene end (bp position)** |
| --- | --- | --- | --- |
| P4HA1 | 1 | 60826989 | 60883912 |
| MYOZ1 | 1 | 61396150 | 61402663 |
| SYNPO2L | 1 | 61406107 | 61412819 |
| VCL | 1 | 61615198 | 61710132 |
| KCNMA1 | 1 | 64178413 | 64684808 |
| ANXA11 | 1 | 66608172 | 66648996 |
| DYNLRB2 | 3 | 28466051 | 28472731 |
| BCO1 | 3 | 29001280 | 29036875 |
| GAN | 3 | 29084755 | 29105590 |
| HSBP1 | 3 | 31167354 | 31168681 |
| DNAAF1 | 3 | 31449251 | 31465094 |
| COTL1 | 3 | 31772229 | 31809779 |
| CDK10 | 3 | 36068115 | 36075811 |
| SPIRE2 | 3 | 36201219 | 36220872 |
| TUBB3 | 3 | 36263694 | 36272042 |
| GAS8 | 3 | 36348525 | 36361935 |
| CENPE | 3 | 36796980 | 36845326 |
| BDH2 | 3 | 36890817 | 36917686 |
| NFKB1 | 3 | 37317740 | 37407632 |
| RAP1GDS1 | 3 | 40810037 | 40927176 |
| FN1 | 6 | 5486218 | 5551290 |
| SMARCAL1 | 6 | 6403081 | 6454865 |
| TNS1 | 6 | 7610232 | 7705182 |
| ARPC2 | 6 | 7918353 | 7949094 |
| AAMP | 6 | 7957551 | 7962161 |
| DYNAP | 8 | 72335561 | 72347793 |
| CCDC68 | 8 | 72623238 | 72668475 |
| SOCS1 | 13 | 33226799 | 33227469 |
| NUBP1 | 13 | 33624865 | 33643241 |
| EMP2 | 13 | 33776335 | 33785814 |
| ATF7IP2 | 13 | 33808727 | 33846726 |
| ABAT | 13 | 35109975 | 35145460 |
| CYFIP2 | 14 | 21808772 | 21911520 |
| SGCD | 14 | 22298803 | 22666954 |
| ACTR1B | 15 | 11610717 | 11618559 |
| ANKRD23 | 15 | 11765031 | 11769143 |
| TEK | 23 | 44907561 | 45009946 |
| C9orf72 | 23 | 45278533 | 45296472 |
| KRT8 | 23 | 47669231 | 47670493 |
| DDX58 | 23 | 49123738 | 49162928 |
| CHMP5 | 23 | 49566805 | 49712668 |
| PALM2-AKAP2 | 25 | 15125991 | 15382273 |
| TXN | 25 | 15434364 | 15444134 |
| TXNDC8 | 25 | 15488256 | 15512774 |
| LPAR1 | 25 | 15923939 | 15976667 |
| INIP | 25 | 17338543 | 17359905 |
| FKBP15 | 25 | 18111748 | 18164311 |
| KIF12 | 25 | 18906427 | 18913478 |
| WHRN | 25 | 19220773 | 19305811 |
| BRWD1 | 26 | 35199604 | 35301887 |

**Blood Flow**

| **Gene symbol** | **Chromosome** | **Gene start (bp position)** | **Gene end (bp position)** |
| --- | --- | --- | --- |
| UNC5B | 1 | 59285358 | 59303802 |
| FAM149B1 | 1 | 60970263 | 61036121 |
| PPP3CB | 1 | 61234363 | 61267642 |
| PLAU | 1 | 61551733 | 61556480 |
| KCNMA1 | 1 | 64178413 | 64684808 |
| DLG5 | 1 | 65017459 | 65144609 |
| ZMIZ1 | 1 | 66286005 | 66392640 |
| CDH13 | 3 | 30386191 | 31158287 |
| SLC38A8 | 3 | 31341377 | 31362222 |
| FOXF1 | 3 | 33360886 | 33363420 |
| CYBA | 3 | 35164679 | 35174016 |
| ANKRD11 | 3 | 35703802 | 35714536 |
| DEF8 | 3 | 36283877 | 36293858 |
| ADGRE2 | 3 | 36485943 | 36493029 |
| PPP3CA | 3 | 38531893 | 38677807 |
| EMCN | 3 | 39159431 | 39170331 |
| ADH1B | 3 | 39988981 | 40069555 |
| RAP1GDS1 | 3 | 40810037 | 40927176 |
| FN1 | 6 | 5486218 | 5551290 |
| XRCC5 | 6 | 6144068 | 6232416 |
| IGFBP5 | 6 | 6609390 | 6625833 |
| AAMP | 6 | 7957551 | 7962161 |
| TMBIM1 | 6 | 7967128 | 7972261 |
| TCF4 | 8 | 72893741 | 73239577 |
| EMP2 | 13 | 33776335 | 33785814 |
| ABAT | 13 | 35109975 | 35145460 |
| ADRA1B | 14 | 19671714 | 19718452 |
| IL12B | 14 | 20194970 | 20203472 |
| EBF1 | 14 | 20400557 | 20778064 |
| FNDC9 | 14 | 21791592 | 21856789 |
| CYFIP2 | 14 | 21808772 | 21911520 |
| SGCD | 14 | 22298803 | 22666954 |
| VWA3B | 15 | 11004018 | 11180724 |
| TMEM131 | 15 | 11323152 | 11499390 |
| SOX11 | 15 | 87001361 | 87002739 |
| DCDC2C | 15 | 88440637 | 88474046 |
| TEK | 23 | 44907561 | 45009946 |
| LINGO2 | 23 | 45593792 | 45595612 |
| TMEM215 | 23 | 49326499 | 49327206 |
| B4GALT1 | 23 | 49574813 | 49594213 |
| AQP3 | 23 | 49837389 | 49842485 |
| ZNF618 | 25 | 18790277 | 18871543 |
| TMEM268 | 25 | 19377520 | 19396657 |
| TNFSF8 | 25 | 19601633 | 19626691 |
| ERG | 26 | 34518620 | 34704944 |

**Hypertension**

| **Gene symbol** | **Chromosome** | **Gene start (bp position)** | **Gene end (bp position)** |
| --- | --- | --- | --- |
| KCNMA1 | 1 | 64178413 | 64684808 |
| CDYL2 | 3 | 28514239 | 28561279 |
| MPHOSPH6 | 3 | 29733789 | 29747437 |
| CDH13 | 3 | 30386191 | 31158287 |
| OSGIN1 | 3 | 31298914 | 31303759 |
| EMC8 | 3 | 32688233 | 32765756 |
| FOXF1 | 3 | 33360886 | 33363420 |
| JPH3 | 3 | 34238255 | 34331020 |
| CYBA | 3 | 35164679 | 35174016 |
| CDH15 | 3 | 35656487 | 35667633 |
| TCF25 | 3 | 36224618 | 36254616 |
| SLC39A8 | 3 | 37578593 | 37625628 |
| MTTP | 3 | 39822153 | 39866768 |
| ADH1B | 3 | 39988981 | 40069555 |
| BMPR1B | 3 | 43301880 | 43858346 |
| CLEC16A | 13 | 33279990 | 33479247 |
| CIITA | 13 | 33499651 | 33545204 |
| ADRA1B | 14 | 19671714 | 19718452 |
| IL12B | 14 | 20194970 | 20203472 |
| SGCD | 14 | 22298803 | 22666954 |
| COX5B | 15 | 11625779 | 11627525 |
| COLEC11 | 15 | 88550817 | 88582392 |
| AQP7 | 23 | 49792490 | 49801137 |
| UBE2R2 | 23 | 49949885 | 49982140 |
| ALAD | 25 | 18298133 | 18302577 |
| ZNF618 | 25 | 18790277 | 18871543 |
| WHRN | 25 | 19220773 | 19305811 |
| GATA3 | 29 | 26022567 | 26039578 |
| PRKCQ | 29 | 27411915 | 27488344 |

**Stroke**

| **Gene symbol** | **Chromosome** | **Gene start (bp position)** | **Gene end (bp position)** |
| --- | --- | --- | --- |
| CDYL2 | 3 | 28514239 | 28561279 |
| PLCG2 | 3 | 29443064 | 29584590 |
| OSGIN1 | 3 | 31298914 | 31303759 |
| EMC8 | 3 | 32688233 | 32765756 |
| FOXF1 | 3 | 33360886 | 33363420 |
| JPH3 | 3 | 34238255 | 34331020 |
| BANP | 3 | 34555030 | 34645020 |
| CYBA | 3 | 35164679 | 35174016 |
| CDH15 | 3 | 35656487 | 35667633 |
| TCF25 | 3 | 36224618 | 36254616 |
| CENPE | 3 | 36796980 | 36845326 |
| NFKB1 | 3 | 37317740 | 37407632 |
| ADH1B | 3 | 39988981 | 40069555 |
| C18orf54 | 8 | 72134316 | 72153014 |
| CLEC16A | 13 | 33279990 | 33479247 |
| CIITA | 13 | 33499651 | 33545204 |
| EBF1 | 14 | 20400557 | 20778064 |
| SGCD | 14 | 22298803 | 22666954 |
| ACTR1B | 15 | 11610717 | 11618559 |
| COX5B | 15 | 11625779 | 11627525 |
| SOX11 | 15 | 87001361 | 87002739 |
| COLEC11 | 15 | 88550817 | 88582392 |
| TEK | 23 | 44907561 | 45009946 |
| LINGO2 | 23 | 45593792 | 45595612 |
| UBE2R2 | 23 | 49949885 | 49982140 |
| WDR31 | 25 | 18243695 | 18253221 |
| ZNF618 | 25 | 18790277 | 18871543 |
| ATP6V1G1 | 25 | 19358809 | 19365266 |
| IGSF5 | 26 | 35670473 | 35700218 |
| GATA3 | 29 | 26022567 | 26039578 |
| SFMBT2 | 29 | 26641660 | 26857577 |
| PRKCQ | 29 | 27411915 | 27488344 |
| PFKFB3 | 29 | 27681953 | 27703408 |
